# Comparison of the baseline characteristics and influencing factors of successful smoking cessation before and during the coronavirus disease pandemic

**DOI:** 10.18332/tid/159237

**Published:** 2023-03-24

**Authors:** Lei Zhu, Rui Zhong, Yanfang Qiu, Jianghua Xie, Yina Hu, Xinhua Yu, Xiaochang Chang, Wei Wang, Lemeng Zhang, Ouying Chen, Hui Cao, Yanhui Zou

**Affiliations:** 1 Hunan Cancer hospital/The Affiliated Cancer Hospital of Xiangya School of Medicine, Central South University, Changsha, China; 2 School of Nursing, Hunan University of Chinese Medicine, Changsha, China; 3 Xiangya Hospital, Central South University, Changsha, China; 4 School of Nursing and Health Management, Wuhan Donghu University, Wuhan, China

**Keywords:** COVID-19, smoking cessation, smoking cessation clinic, smoking cessation rate, predictors

## Abstract

**INTRODUCTION:**

Travel and living environment restrictions, which may have positive or negative effects on smoking-related behaviors, were implemented to limit the COVID-19 pandemic. This study aimed to compare the baseline clinical characteristics and smoking cessation (SC) rate at 3 months of patients in an SC clinic in Hunan Province, China before and during the COVID-19 pandemic and identify influencing factors of successful SC.

**METHODS:**

Healthy patients at the SC clinic aged ≥18 years before the COVID-19 pandemic and during the COVID-19 pandemic were divided into groups A and B, respectively. The two groups’ demographic data and smoking characteristics were compared, and SC interventions were applied by the same medical staff team through telephone follow-up and counselling during the SC procedure.

**RESULTS:**

Groups A and B included 306 and 212 patients, respectively, with no significant differences in demographic data. The SC rates of group A (pre COVID-19) and group B (during the COVID-19 pandemic) at 3 months were 23.5% and 30.7%, respectively, after the first SC visit. Those who chose to quit immediately or within 7 days were more successful than those who did not choose a quit date (p=0.002, p=0.000). Patients who learned about the SC clinic via network resources and other methods were more likely to succeed than those who learned about the clinic from their doctor or hospital publications (p=0.064, p=0.050).

**CONCLUSIONS:**

Planning to quit smoking immediately or within 7 days of visiting the SC clinic and learning about the SC clinic via the network media or other methods improved the likelihood of successful SC. SC clinics and the harm of tobacco should be promoted via network media. During consultation, the smokers should be encouraged to quit smoking immediately and establish an SC plan, which would help them to quit smoking.

## INTRODUCTION

Despite a decline in the smoking rates in China in recent years, that among people aged ≥15 years is as high as 26.6%, and only 16.1% of current smokers (CS) plan to quit in the next 12 months^1^.

From early 2020 until the present, the coronavirus disease 2019 (COVID-19) pandemic has been a major public health event, significantly affecting people’s livelihoods worldwide^2^. Previously, studies regarding the relationship between COVID-19 and smoking, and changes in smoking behavior due to COVID-19, have been reported. Patanavanich et al.^3^ reported that susceptibility to and the progression of COVID-19 were associated with smoking . A meta-analysis of 11322 patients with COVID-19 confirmed that smoking was associated with severe COVID-19 disease in current and previous smokers^4^. Other studies reported that smoking is associated with the severity of COVID-19^5,6^.

Studies have also confirmed that the prevalence of COVID-19 is higher among smokers. Active smoking upregulates the expression of Angiotensin-converting enzyme 2 (ACE2) in the respiratory tract, increasing the risk of severe COVID-19 infection^7^. The World Health Organization advises patients to quit smoking, and states that increased smoking cessation (SC) rates may reduce the risk of infectious diseases^8^. Previous studies have reported that SC can normalize parts of the respiratory epithelial structure, reduce proliferation, down-regulate ACE2 levels, and improve endothelial function^9^. However, some patients have misconceptions regarding smoking and COVID-19, believing that smoking can prevent COVID-19 infection or that tobacco smoke has bactericidal and anti-viral effects^10^.

To help control the COVID-19 pandemic, most countries enforced travelling and living environment restrictions. In addition, SC rates, smoking-related behaviors, and intention to quit rates have changed during the COVID-19 pandemic^11^. Kayhan et al.^12^ reported an increase in the SC rate from 2019 (23.7%) to 2020 (31.1%). Fear of COVID-19 infection was cited as a reason to quit by 46.2% (165/357) of the participants, and the success of quitting smoking was not significantly associated with age, sex, drug use, or nicotine addiction score.

A study conducted in the Netherlands reported that 14.1% of smokers reported smoking less due to the COVID-19 pandemic, and 18.9% of smokers reported smoking more; the most commonly reported reason for reducing smoking was to live a healthy lifestyle, whereas the reason for increasing smoking was cited as boredom and high stress, which may have been caused by the COVID-19 pandemic^13^. Boredom and loneliness have been reported as common reasons to increase smoking behavior, and the threat of COVID-19 infection has been reported as a reason to reduce or quit smoking.

However, studies have yet to report on the changes in smoking characteristics and intention to quit before and during the COVID-19 pandemic. Therefore, this study analyzed the characteristics of patients at their first visit to an SC clinic in a tertiary hospital before and during the COVID-19 pandemic. The SC rate at 3 months was compared between patients, and factors influencing SC before and during the COVID-19 pandemic were compared.

## METHODS

### Study design and participant recruitment

Participants who intended to quit smoking via an SC clinic at a tertiary hospital before the COVID-19 pandemic (first visit: 1 September 2015 to 15 December 2019) were included in group A. Participants who intended to quit smoking during the COVID-19 pandemic (first visit: 4 January to 12 November 2021) were included in group B. Both groups were provided SC guidance and counselling and were followed-up by trained medical staff from the same SC clinic.

All participants were aged ≥18 years (smoked ≥1 cigarette/day, for more than 6 months), in good health, and were willing to quit smoking. Smartphones were used to contact all participants. Participants with severe or life-threatening diseases, cancer, or cognitive dysfunction, and those with missing data, were excluded. Informed consent was obtained from all participants. This study was approved by the ethics committee of Hunan Cancer Hospital and was conducted in accordance with the Declaration of Helsinki.

### Data collection and evaluation indices

Participants’ baseline data and smoking status were collected during the initial visit using the SC clinic questionnaire^14^ based on the guidelines for SC clinic practices. Data on participant sex, age, education level, occupation, daily consumption of cigarettes, smoking duration (year), and planned quit date were collected. The exhaled carbon monoxide (CO) test was conducted at the first visit. The included participants were categorized as non-smokers, light smokers, and heavy smokers based on the CO test results. A CO of 0–6 ppm was indicative of not smoking, while 7–10 ppm was indicative of light smoking. Participants who scored 11–72 ppm were considered heavy smokers^15^.

The exhaled CO concentration is related to the average number of cigarettes smoked daily and the duration of smoking. When the average number of cigarettes smoked per day is less than 10 or the duration of smoking is less than 5 years, the exhaled CO concentration may still be within the normal range of 0–6 ppm^16^, so CO test cannot be used as a basis for determining the success of smoking cessation. In addition, considering the time and space limitations of smoking cessation clinics and the impact of the COVID-19 pandemic, self-reported smoking cessation was defined as an indicator to confirm successful smoking cessation.

The Fagerström Test for Nicotine Dependence (FTND)^17^, a six-question survey, was used to assess nicotine dependence. A score of 0 was considered no dependence, 1–3 as low dependence, 4–6 as moderate dependence, and 7–10 as high dependence.

The primary outcome measures were the SC rate at 1 and 3 months. Successful quitting of smoking was decided based on the self-report of smokers who have maintained their smoking cessation status from the beginning of treatment until the end. Participants who gave wrong phone numbers and those who responded to our phone calls less than seven times during the study were considered lost to follow-up.

### Smoking cessation interventions

Patients were offered face-to-face behavioral support and three standardized telephone follow-up and support visits^18-20^ at 1 week, 1 month, and 3 months. SC medications were offered to participants at their own expense.

### Statistical analysis

Univariate analysis of SC at 3 months was conducted using the chi-squared test. Data that were not normally distributed were compared using the rank sum test. A binary logistic regression analysis was used to identify factors that predicted success at 3 months. Unadjusted odds ratios (ORs) and 95% confidence intervals (CIs) were evaluated using a multivariable analysis with α_in_=0.05 and α_out_=0.1. All analyses were conducted using SPSS version 23.0 (SPSS, Chicago, IL, USA). Statistical significance was set at p<0.05.

## RESULTS

### Demographic data

Group A included 329 participants and group B included 249. After excluding 23 participants in group A and 37 in group B for poor health, the final sample comprised 306 participants in group A and 212 in group B. The participant’s age, sex, education level, and occupation were not significantly different between the two groups. Overall, the median participant age was 46 years (18–75 years) (Table 1).

**Table 1:** Comparison of demographic information between the pre COVID-19 and during COVID-19 participants (N=518)

*Variables*	*Total (N=518)*	*Pre COVID-19 (A group) (N=306)*	*During COVID-19 (B group) (N=212)*	*χ^2^/Z*	*p*
	*n (%)*	*n (%)*	*n (%)*		
**Gender**				1.118	0.290
Male	499 (96.3)	297 (97.1)	202 (95.3)		
Female	19 (3.7)	9 (2.9)	10 (4.7)		
**Age** (years), mean ± SD		44.85 ± 11.77	45.91 ± 12.54	-1.021	0.307
<45	224 (43.2)	140 (45.8)	84 (39.6)		
45–59	212 (40.9)	127 (41.5)	85 (40.1)		
≥60	82 (15.8)	39 (12.7)	43 (20.3)		
**Education level**				6.695	0.082
Primary school or lower	55 (10.6)	24 (7.8)	31 (14.6)		
Middle school	156 (30.1)	94 (30.7)	62 (29.2)		
High school	136 (26.3)	80 (26.1)	56 (26.4)		
College school or higher	171 (33.0)	108 (35.3)	63 (29.7)		
**Occupation**				3.176	0.365
Farmers	126 (24.3)	74 (24.2)	52 (24.5)		
Workers	311 (60.0)	181 (59.2)	130 (61.3)		
Retired/unemployed	28 (5.4)	21 (6.9)	7 (3.3)		
Other	53 (10.2)	30 (9.8)	23 (10.8)		

A group: Participants who intended to quit smoking via an SC clinic of a tertiary hospital in Hunan, China, before the COVID-19 pandemic (first visit: 1 September 2015 to 15 December 2019). B group: Participants who intended to quit smoking via an SC clinic of a tertiary hospital in Hunan, China, during the COVID-19 pandemic (first visit: 4 January to 12 November 2021). SC: smoking cessation. SD: standard deviation.

In group A, 97.1% (297/306) of participants were males, and the average age was 44.85 ± 11.77 years. The education level of group A was mainly college level or higher (35.3%; 108/306), although 30.7% (94/306) reported a middle school level education. The majority of participants in group A (59.2%) were workers (in this study, ‘workers’ mainly refers to manual employers in the city, usually not working in an office).

In group B, 95.3% (202/212) of participants were males. The average age was 45.91 ± 12.54 years, and 61.3 % were workers.

### Smoking characteristics

The average number of smoking years in group A was 22.26 ± 11.96 years, and the mean number of cigarettes/day smoked was 22.24 ± 11.13. Most participants in group A (30.1%; 92/306) smoked for 11–20 years, whereas 52.9% (162/306) smoked 11–20 cigarettes per day. In group A, 77.2% of participants smoked >10 years; the longest smoking time was 60 years.

In group B, the average number of smoking years was 24.11 ± 12.78 years, and the mean number of cigarettes/day smoked was 25.11 ± 13.58. Sixty participants (28.3%) smoked for 21–30 years, whereas 78.8% (167/212) smoked for more than ten years. The longest smoking time reported in group B was 50 years. The number of smoking years and daily smoking volume were not significantly different between the groups (Table 2).

**Table 2:** Clinical characteristic of participants between the pre COVID-19 and during COVID-19 participants (N=518)

*Variables*	*Total (N=518)*	*Pre COVID-19 (A group) (N=306)*	*During COVID-19 (B group) (N=212)*	*χ^2^/Z*	*p*
	*n (%)*	*n (%)*	*n (%)*		
**Duration of smoking** (years), mean ± SD		22.26 ± 11.961	24.11 ± 12.783	-0.836	0.403
≤10	115 (22.2)	70 (22.9)	45 (21.2)		
11–20	150 (29.0)	92 (30.1)	58 (27.4)		
21–30	145 (28.0)	85 (27.8)	60 (28.3)		
≥31	108 (20.8)	59 (19.3)	49 (23.1)		
**Daily cigarettes consumption,** mean ± SD		22.24 ± 11.128	25.11 ± 13.576	-0.206	0.837
≤10	93 (18.0)	53 (17.3)	40 (18.9)		
11–20	242 (46.7)	162 (52.9)	80 (37.7)		
≥21	183 (35.3)	91 (29.7)	92 (43.4)		
**SC attempts previously**				3.406	0.065
No	217 (41.9)	118 (38.6)	99 (46.7)		
Yes	301 (58.1)	188 (61.4)	113 (53.3)		
**Fagerström score,** mean ± SD		4.32 ± 2.049	5.08 ± 2.918	-0.237	0.813
≤3 (mild)	170 (32.8)	106 (34.6)	64 (30.2)		
4–6 (moderate)	231 (44.6)	157 (51.3)	74 (34.9)		
≥7 (severe)	117 (22.6)	43 (14.1)	74 (34.9)		
**CO test results** (ppm)				10.410	0.005
0–6	93 (27.6)	31 (20.3)	62 (33.7)		
7–10	55 (16.3)	22 (14.4)	33 (17.9)		
11–72	189 (56.1)	100 (65.4)	89 (48.4)		
**Planned quit date**				66.349	0.000
Undecided	127 (24.5)	62 (20.3)	65 (30.7)		
Immediately	141 (27.2)	80 (26.1)	61 (28.8)		
Within 7 days	68 (13.1)	22 (7.2)	46 (21.7)		
Within 30 days	133 (25.7)	93 (30.4)	40 (18.9)		
Over 30 days	49 (9.5)	49 (16.0)	0 (0.0)		
**SC adds**					0.121*
BS	495 (95.6)	292 (95.4)	203 (95.8)		
BS and medication	18 (3.5)	9 (2.9)	9 (4.2)		
BS and EC	5 (1.0)	5 (1.6)	0 (0.0)		
**Method of learning about the SC clinic**				66.491	0.000
Medical staff and hospital publicity	371 (71.6)	252 (82.4)	119 (56.1)		
Network media	45 (8.7)	30 (9.8)	15 (7.1)		
Family informed	52 (10.0)	13 (4.2)	39 (18.4)		
Other	50 (9.7)	11 (3.6)	39 (18.4)		

BS: behavior support. EC: electronic cigarettes.

* Monte Carlo p. A group: Participants who intended to quit smoking via an SC clinic of a tertiary hospital in Hunan, China, before the COVID-19 pandemic (first visit: 1 September 2015 to 15 December 2019). B group: Participants who intended to quit smoking via an SC clinic of a tertiary hospital in Hunan, China, during the COVID-19 pandemic (first visit: 4 January to 12 November 2021). SC: smoking cessation. SD: standard deviation.

### Nicotine dependence score and CO test results

The overall median FTND score was 5 points, and the average FTND score was 4.32 ± 2.05 points in group A and 5.08 ± 2.92 points in group B (p=0.813).

A total of 337 participants underwent the CO test (CO tests were performed according to the participants’ wishes, and we performed CO tests only for participants willing to accept the procedure). Most participants were heavy smokers, including 65.4% (100/153) of participants in group A and 48.4% (89/184) of participants in group B (p=0.005) (Table 2).

### Planned quit date and smoking cessation rate

In group A, after visiting the SC clinic, 26.1% (80/306) of participants planned to quit smoking immediately, 7.2% (22/306) planned to quit smoking within seven days, 30.4% (93/306) planned to quit smoking within 30 days, 16.0% (49/306) planned to quit smoking after 30 days, and 20.3% (62/306) did not have a planned quit date (no planned quit date was considered ‘undecided’). In group B, after visiting the SC clinic, 28.8% (61/212) of participants planned to quit smoking immediately, 21.7% (46/212) planned to quit smoking within seven days, 18.9% (40/212) planned to quit smoking within 30 days, no participants planned to quit smoking after 30 days, and 30.7% (65/212) did not have a planned quit date. The planned quit date was significantly different between the groups (p=0.000).

During the 3-month follow-up period, 95.4% (292/306) of the participants in group A received behavioral support to quit smoking, 2.9% (9/306) received SC medication and behavioral support, and 1.6% (5/306) used electronic cigarettes and behavioral support to quit smoking.

In group B, 95.8% (203/212) of the participants received behavioral support, 4.2% (9/212) received SC medication and behavioral support, and no patients used electronic cigarettes. The SC methods were not significantly different between the two groups (p=0.121) (Table 2).

### Access to smoking cessation clinic information

Participants in group A reported learning about the SC clinic from the medical staff, hospital publications (82.4%), and network media (9.8%). Participants in group B reported learning about the SC clinic from the medical staff, hospital publications (56.1%), family members (18.4%), and other means (18.4%). How the participants learned about the SC clinic differed between the groups (p=0.000) (Table 2).

### Smoking cessation rate and clinical characteristics at three months

The SC rate at 3 months was 23.5% (72/306) in group A and 30.7% (65/212) in group B. In terms of years of smoking duration (≤10 years, 21–30 years, ≥31 years), the number of cigarettes smoked daily, degree of nicotine dependence, and CO test results, and the proportion of smokers in group B who planned to quit smoking within three months was higher than that in group A. When smokers who did not choose a planned quit date were excluded, the SC rate remained higher in group B (28.8%) than in group A (19.3%). The SC rate among patients who did not choose a planned quit date or chose a planned quit date more than 30 days after the first visit to the SC clinic was lower in group B (undecided: 6.2%; >30 days: 0.0%) than in group A (undecided: 21.0%; >30 days: 2.0%). The SC rate among patients who planned to quit smoking immediately or within seven days of the first visit to the SC was higher in group B (immediately: 50.8%; within seven days: 45.7%) than in group A (immediately: 33.8%; within seven days: 40.9%) (Table 3).

**Table 3:** Clinical characteristic the pre COVID-19 and during COVID-19 participants in smoking cessation for three months (N=518)

*Variables*	*Total (N=518) n (%)*	*Pre COVID-19 (A group) (N=306)*				*During COVID-19 (B group) (N=212)*			
		*Abstinence failed (N=234) n (%)*	*Abstinence succeeded (N=72) n (%)*	*χ^2^*	*p*	*Abstinence failed (N=147) n (%)*	*Abstinence succeeded (N=65) n (%)*	*χ^2^*	*p*
**Duration of smoking** (years)				2.144	0.543			1.843	0.606
≤10	115 (22.2)	50 (71.4)	20 (28.6)			30 (66.7)	15 (33.3)		
11–20	150 (29.0)	69 (75.0)	23 (25.0)			44 (75.9)	14 (24.1)		
21–30	145 (28.0)	67 (78.8)	18 (21.2)			39 (65.0)	21 (35.0)		
≥31	108 (20.8)	48 (81.4)	11 (18.6)			34 (69.4)	15 (30.6)		
**Daily cigarettes consumption**				1.025	0.599			4.121	0.127
≤10	93 (18.0)	38 (71.7)	15 (28.3)			25 (62.5)	15 (37.5)		
11–20	242 (46.7)	127 (78.4)	35 (21.6)			62 (77.5)	18 (22.5)		
≥21	183 (35.3)	69 (75.8)	22 (24.2)			60 (65.2)	32 (34.8)		
**SC attempts previously**				7.310	0.007			0.163	0.686
No	217 (41.9)	100 (84.7)	18 (15.3)			70 (70.7)	29 (29.3)		
Yes	301 (58.1)	134 (71.3)	54 (28.7)			77 (68.1)	36 (31.9)		
**Fagerström score**				2.053	0.358			3.378	0.185
≤3 (mild)	170 (32.8)	76 (71.7)	30 (28.3)			43 (67.2)	21 (32.8)		
4–6 (moderate)	231 (44.6)	124 (79.0)	33 (21.0)			57 (77.0)	17 (23.0)		
≥7 (severe)	117 (22.6)	34 (79.1)	9 (20.9)			47 (63.5)	27 (36.5)		
**CO test results** (ppm)					0.841*			3.750	0.153
0–6	93 (27.6)	26 (83.9)	5 (16.1)			38 (61.3)	24 (38.7)		
7–10	55 (16.3)	17 (77.3)	5 (22.7)			21 (63.6)	12 (36.4)		
11–72	189 (56.1)	78 (78.0)	22 (22.0)			67 (75.3)	22 (24.7)		
**Planned quit date**				21.140	0.000			36.138	0.000
Undecided	127 (24.5)	49 (79.0)	13 (21.0)			61 (93.8)	4 (6.2)		
Immediately	141 (27.2)	53 (66.3)	27 (33.8)			30 (49.2)	31 (50.8)		
Within 7 days	68 (13.1)	13 (59.1)	9 (40.9)			25 (54.3)	21 (45.7)		
Within 30 days	133 (25.7)	71 (76.3)	22 (23.7)			31 (77.5)	9 (22.5)		
Over 30 days	49 (9.5)	48 (98.0)	1 (2.0)			0 (0.0)	0 (0.0)		
**SC adds**					0.063*			1.654^a^	0.198
BS	495 (95.6)	226 (77.4)	66 (22.6)			143 (70.4)	60 (29.6)		
BS and medication	18 (3.5)	4 (44.4)	5 (55.6)			4 (44.4)	5 (55.6)		
BS and EC	5 (1.0)	4 (80.0)	1 (20.0)			0 (0.0)	0 (0.0)		
**Method of learning about the SC clinic**					0.611*				0.014*
Medical staff and hospital publicity	371 (71.6)	193 (76.6)	59 (23.4)			90 (75.6)	29 (24.4)		
Network media	45 (8.7)	21 (70.0)	9 (30.0)			7 (46.7)	8 (53.3)		
Family informed	52 (10.0)	12 (92.3)	1 (7.7)			30 (76.9)	9 (23.1)		
Other	50 (9.7)	8 (72.7)	3 (27.3)			20 (51.3)	19 (48.7)		

a Continuous correction value. BS: behavior support, EC: electronic cigarettes,

* Monte Carlo p. A group: Participants who intended to quit smoking via an SC clinic of a tertiary hospital in Hunan, China, before the COVID-19 pandemic (first visit: 1 September 2015 to 15 December 2019). B group: Participants who intended to quit smoking via an SC clinic of a tertiary hospital in Hunan, China, during the COVID-19 pandemic (first visit: 4 January to 12 November 2021). SC: smoking cessation.

### Predictors of successful smoking cessation at three months

Participant group, CO test results, planned quit date, and access to SC clinic information were the independent variables used in the binary multivariate logistic regression analysis. The influencing factors of successful SC were not significantly different between the groups (p=0.856). The probability of successful SC was 4.88 times higher for patients who planned to quit smoking immediately (OR=4.88; 95% CI: 1.79–13.33; p=0.002) and 6.38 times higher for patients who planned to quit smoking within seven days (OR=6.38; 95% CI: 2.28–17.81; p=0.000) than those who did not choose a planned quit date. The SC rate was 2.35 times higher among participants who learned about the SC clinic from network media (OR=2.35; 95% CI: 0.95–5.78; p=0.064) and 2.20 times higher among participants who learned about the SC clinic via other ways (OR=2.20; 95% CI: 1.00– 4.84; p=0.050) than those who learned about the SC clinic from the medical staff or hospital publications (Table 4).

**Table 4:** Predictive factors for smoking cessation as identified through the binary logistic regression analysis

*Variables*	*β*	*SE*	*Wald χ^2^*	*p*	*OR*	*95 % CI*
**Group**						
A (Ref.)					1	
B	0.058	0.319	0.033	0.856	1.06	0.57–1.98
**CO test results** (ppm)						
0–6 (Ref.)					1	
7–10	-0.047	0.410	0.013	0.908	0.95	0.43–2.13
11–72	-0.127	0.315	0.163	0.686	0.88	0.48–1.63
**Planned quit date**						
Undecided (Ref.)					1	
Immediately	1.586	0.512	9.577	0.002	4.88	1.79–13.33
Within 7 days	1.853	0.524	12.488	0.000	6.38	2.28–17.81
Within 30 days	0.841	0.533	2.486	0.115	2.32	0.82–6.59
After 30 days	-19.051	7153.985	0.000	0.998	0.00	0.00–0.00
**Method of learning about the SC clinic**						
Medical staff and hospital publicity (Ref.)					1	
Network media	0.852	0.460	3.436	0.064	2.35	0.95–5.78
Family informed	0.310	0.468	0.439	0.508	1.36	0.55–3.41
Other	0.788	0.403	3.834	0.050	2.20	1.00–4.84

## DISCUSSION

In this study, fewer heavy smokers sought SC services during the pandemic. Before and during COVID-19, smokers had different means of learning about SC clinics and planning to quit immediately or within seven days was a predictor of SC success in smokers.

### Planned quit date as a predictor of successful smoking cessation

The influence of the planned quit date on SC success was significantly different before and during the COVID-19 pandemic. During the COVID-19 pandemic, the probability of successful SC was higher among patients who planned to quit immediately and those who planned to quit within seven days. In our study, during the COVID-19 pandemic, 18.9% of participants planned to quit smoking within one month of their first visit to an SC clinic, and no participant planned to quit smoking after 30 days of visiting the SC clinic. Elling et al.^21^ reported that 36.5% (124/340) of CSs who intended to quit in the five years before the COVID-19 pandemic were willing to quit smoking within six months and 18.5% (63/340) were willing to quit smoking within one month. In addition, 33.8% of the survey respondents were highly motivated to quit smoking because of the COVID-19 pandemic. Our results were consistent with the findings of Elling et al.^22^. Thus, it follows that a higher proportion of smokers intended to start quitting early during the COVID-19 pandemic and this intention increased during the COVID-19 pandemic. The planned quit date was related to the intention to quit smoking; the earlier the planned quit date, the stronger the intention to quit.

A strong intention to quit smoking and previous attempts to quit smoking have been reported as key factors to successful SC. The results of the current study are consistent with these previous findings. The participants’ intention to quit and the number of attempts increased during the pandemic, which may reflect concern regarding COVID-19 infection^23^. Dutcher et al.^24^ reported a positive correlation between the number of SC attempts and the current motivation to quit smoking (r=0.17; 95% CI: 0.14– 0.20). Nan et al.^25^ investigated the intention to quit smoking among Chinese participants aged ≥15 years in 2018 and reported that 17.96% of CSS had tried to quit smoking in the past 12 months, and 6.63% of CSs planned to quit within one month. According to a national survey conducted by Xie et al.^26^, 17.6% of CSs in China considered starting to quit smoking in the next 12 months. Smokers who smoked more cigarettes per day (one pack or more) had lower intentions of quitting smoking, and smokers with a lower degree of tobacco dependence had higher intentions of quitting smoking. Therefore, the intention to quit smoking is low among CSs, and the government should actively advocate for the public to quit smoking through multiple channels, which may reduce the smoking volume and encourage SC, ultimately improving the SC rate.

### Heavy smokers seeking smoking cessation services decreased during the pandemic

The CO breath analyzer test is used to monitor the concentration of CO in patients’ exhaled breath and evaluate the progress of SC. Hu et al.^19^ reported that before the pandemic, 80.7% of participants who underwent the CO test in an SC clinic in Hunan, China, were heavy smokers, and 19.3% were light smokers. In the study results of Xie et al.^20^, 15.5% of the participants who underwent the CO test were light smokers, and 63.2% were heavy smokers. This is similar to the results of the present study. In the current study, among participants who underwent the CO test in SC clinics before the pandemic, 14.4% were light smokers, and 65.4% were heavy smokers. During the COVID-19 pandemic, 17.9% were light smokers, and 48.4% were heavy smokers. It follows that more light smokers sought SC services and fewer heavy smokers sought SC services during the pandemic. The exhaled CO concentration is related to the average number of cigarettes smoked daily and the duration of smoking. With the increase in the daily amount and duration of smoking, the exhaled CO concentration of smokers also showed an increasing trend^16^. Mao et al.^10^ found a decrease in the number of cigarettes smoked per day, from 14.2 cigarettes/day before the pandemic to 13.5 cigarettes/day during the pandemic, owing to smokers being more concerned about their health during the pandemic and the inconvenience of wearing masks when smoking. Yu et al.^27^ also reported that 32.3% (7266/22459) of frontline epidemic prevention workers in China were willing to reduce or stop smoking owing to the COVID-19 pandemic. The decrease in the average number of cigarettes smoked per day by smokers may be why more light smokers sought SC services, and fewer heavy smokers sought SC services during the pandemic. Matuszewski et al.^28^ reported that 71% of CSs increased their intention to quit after using a CO detector, and 40% had consulted a quit helpline. The exhaled CO level assesses changes in the smoking volume, helping SC workers understand the participants’ status and adjust the intervention as needed.

### Methods of learning about smoking cessation clinics differed before and during COVID-19

Participants learned about SC clinics differently before and during the COVID-19 pandemic. Before the pandemic, participants learned about SC clinics via the medical staff, hospital publications and online media information. This may be related to increased social media use by smokers during the COVID-19 pandemic. Li et al.^29^ reported that Chinese ‘netizens’ (Internet users) spent an average of 2–3 hours per day (average 2.34 hours, SD=1.11) using social media during the COVID-19 pandemic. During the COVID-19 pandemic, a lower proportion of participants learned about SC clinics from the medical staff and hospital publications, although more than half of the participants still reported this method. The proportion of participants who learned about SC clinics from their family members increased significantly during the pandemic, and the study by O’Donnell et al.^30^ may explain why this occurred. O’Donnell et al.^30^ conducted semi-structured interviews with adults aged >21 years during the COVID-19 pandemic and found positive and negative effects on smoking behaviors. The interviewees reported an inconvenience in smoking, resulting in reduced smoking. Family members of smokers were more concerned about the harm of smoking behaviors, resulting in smokers being more willing to seek other healthy activities^31^. Regidor et al.^32^ reported that the willingness to quit and quit rates increased in smoke-free environments. Tobacco control and SC route promotion are related to the intention to quit smoking. Previous studies reported that CSs who consulted a doctor in the previous 12 months and were advised to quit were more likely to quit smoking than smokers who had not visited a doctor in the previous 12 months^33^. CSs who obtained tobacco control information from any media source within 30 days or had visited hospitals within 12 months and received medical advice from medical staff regarding quitting smoking had higher intentions to quit smoking^34^. Participants who had received medical advice and information about smoking control online were also more willing to quit smoking. Another study reported that smokers who received SC advice or support from their primary doctor were more likely to have tried to quit than those who did not receive advice^35^.

A study in the Netherlands reported that tobacco control policies and support for SC positively affected the attempt to quit smoking and SC rates^36^. Previous studies reported that the intention to quit was higher when smoking was not permitted in the home, which may be due to the inconvenience of smoking or the increased awareness of the dangers of smoking among family members^26^. Smokers with average or poor cognition regarding the dangers of smoking were less likely to quit smoking and more likely to smoke than those with superior awareness. Currently, medical staff and hospital publications are the primary way by which individuals learn about SC clinics. Therefore, SC clinics and tobacco control departments should increase publicity regarding SC clinics and tobacco harm and improve the awareness of SC among smokers and their families.

### Limitations

This study has some limitations. SC success was determined based on self-reported results during follow-up telephone calls, which may have biased the results. Future multi-center studies with larger sample sizes are needed to clarify the effects of the COVID-19 pandemic on SC.

## CONCLUSIONS

During the COVID-19 pandemic, information regarding SC clinics was available to smokers, mostly from family members compared to before the pandemic, and the willingness to smoke increased during the pandemic. The probability of successful SC in 3 months increased among participants who planned to quit smoking immediately or within 7 days of visiting the SC clinic. Government publications regarding SC clinics and the dangers of tobacco use helped individuals plan to quit smoking. It is important to establish an SC plan, especially for smokers to quit immediately, which ultimately improves the SC rate.

## Data Availability

The data supporting this research are available from the authors on reasonable request.
